# A database of seed plants on taxonomy, geography and ecology in the Qinling-Daba Mountains and adjacent areas

**DOI:** 10.1038/s41597-022-01810-8

**Published:** 2022-11-04

**Authors:** Xinghang Zhang, Baiping Zhang, Jiayu Li, Yonghui Yao, Jing Wang, Junjie Liu, Fuqin Yu

**Affiliations:** 1 State Key Laboratory of Resources and Environmental Information System, Institute of Geographic Sciences and Natural Resources Research, Chinese Academy of Sciences, Beijing, 100101 China; 2 University of Chinese Academy of Sciences, Beijing, 100049 China

**Keywords:** Biodiversity, Forest ecology, Biogeography

## Abstract

The Qinling-Daba Mountains span subtropical and warm temperate zones and are one of the most remarkable biodiversity hotspots in China. Establishing a complete checklist of seed plants organized by nature reserves in the Qinling-Daba Mountains and adjacent areas is an important basis for managing and utilizing plant resources. First, we collected seed plant species data from published checklists representing 58 nature reserves in the Qinling-Daba Mountains and adjacent areas; second, we comprehensively and systematically sorted and integrated these data; third, we proofread and revised the data with the help of the R language and *Flora of China dataset*; and finally, we set up a seed plant database containing 96148 records, including the name, order, family, genus, life form, and endemism of each species for the entirety of the Qinling-Daba Mountains. The database contains 9491 species of seed plants belonging to 1729 genera, 211 families, and 59 orders, accounting for 39% of China’s seed plants.

## Abstract (summary)

**Table:** 

Measurement(s)	seed plants on taxonomy, geography and ecology
Technology Type(s)	literature search, data extraction and structure
Sample Characteristic - Organism	Spermatophyta
Sample Characteristic - Environment	mountain range
Sample Characteristic - Location	Qinling Mountains • Daba Mountains

## Background & Summary

The Qinling-Daba Mountains are not only a complete geographical and geomorphic unit but are also a natural boundary dividing the geographic and climatic conditions between northern and southern China. The mountains are also a bridge connecting the Qinghai-Tibet Plateau and the eastern plain and an important boundary between the warm temperate zone and the subtropical zone^1^. This region is thus of great importance for the formation of geographical patterns, the evolution of biota and the distribution of natural resources in China^2,3^. The Qinling-Daba Mountains are biodiversity hotspots in China; more than 2500 species of higher plants live in many mountainous areas within the range, such as the Funiu Mountains and Shennongjia Mountains^4,5^. Throughout the Qinling-Daba Mountains, the proportion of species that are endemic to China is extremely high, exceeding 1/3 of the total number of higher plant species found in the region. The regional differences and distribution patterns of biological species are closely related to the characteristics of the mountain environments in different mountainous areas. It is necessary to understand the distribution pattern of biodiversity in the Qinling-Daba Mountains based on the structure and uniqueness of the geographical environment to not only provide a more solid scientific foundation for national biodiversity protection but also contribute to global biodiversity research.

As a “mirror” reflecting the natural geographical environment, plants are an important embodiment of complex mountainous environments. At the same time, plants are among the most important resources in nature. Mastering the properties, characteristics and development patterns of plants can provide a scientific basis for local agricultural activities, forestry, pastoral areas and production and construction. The ecological factors of plants in the Qinling-Daba Mountains are mainly affected by multidimensional zonality, such as vertical zonality, latitudinal zonality and aspect differences, features enhance the diversity, complexity and uniqueness of mountain plants. However, due to land use changes, intensive agricultural activities and diseases, some plant species in the Qinling-Daba Mountains may be slowly decreasing^6^. In addition to the impacts of these human activities, available information regarding most wild seed plant species in the Qinling-Daba Mountains is sporadic and fragmented, and the understanding of endemic seed plant species in the Qinling-Daba Mountains is insufficient. This plant information gap also poses a major threat to the protection of these plants. As a geographical transition zone and ecological ecotone, some scholars believe that a competition mechanism is at play among plants in the Qinling-Daba Mountains^7^; this mechanism may reduce the plant diversity in the mountains and render general protection strategies insufficient. In this sense, obtaining a plant species database is very important, as it can serve as a source of natural and historical heritage information and can be used to conduct new research, evaluate species’ protection statuses, and provide information for decision-making.

In the past, research on plants in the Qinling-Daba Mountains was mainly focused on local areas (provinces), and scattered and sporadic investigative work was carried out, especially local biological investigations. To date, no comprehensive scientific investigation or research has been organized. The key basic geographic data reveal obvious “fragmentation” characteristics; the data are spatially incomplete and difficult to connect and integrate, resulting in a weak foundation for scientific research in this region. Therefore, it is urgent to conduct systematic sorting to obtain basic seed plant data for the entirety of the Qinling-Daba Mountains and deepen our comprehensive understanding of the north–south transition zone. However, our ecological and behavioral knowledge of plants in this region is very limited. In some cases, this knowledge is anecdotal. At present, the plant species in the Qinling-Daba Mountains are known to basically present local and regional species diversity, including in the mountains of western Henan Province, southern Gansu Province, the Qinling Mountains in Shaanxi Province and the Daba Mountains in Sichuan Province, Chongqing Province and Hubei Province^5,8–12^. In view of the geographical scope and biogeographic background of the Qinling-Daba Mountains, these mountains and their adjacent areas support nature reserves at many different administrative levels, such as national nature reserves, provincial nature reserves and county-level nature reserves; these reserves host a great diversity of plant species and a high number of endemic species, especially in national nature reserves. The warm temperate and subtropical transitional mountainous climate of the Qinling-Daba Mountains contributes to this global biodiversity hotspot, and the region is among the mountainous areas with the most endemic seed plant species in China^13^.

Establishing a complete checklist of the seed plants present in the nature reserves in the Qinling-Daba Mountains and adjacent areas, including the scientific names, taxa, life forms, geographical distributions and endemism of plant species, is not only important for mastering and utilizing national plant resources but is also a key foundation for the development of research in geography, ecology and other relevant subjects. Unfortunately, the currently available data collected in nature reserves are very scattered and dispersed, and our knowledge of seed plants and ecology in the Qinling-Daba Mountains is rather sparse, posing a major threat to the conservation of plants and the ecological environment. Here, we first collected published seed plant checklists representing 58 nature reserves in the Qinling-Daba Mountains and adjacent areas. Second, we comprehensively and systematically sorted and integrated these data. Third, we proofread and revised the data in combination with the R language and *Flora of China* (http://www.iplant.cn/). Finally, we obtained a database containing 96148 records, including the name, taxonomy, life form and endemism information of each seed plant species in the entirety of the Qinling-Daba Mountains. The database contains 9491 seed plant species belonging to 1729 genera, 211 families, and 59 orders, accounting for 39% of Chinese seed plant diversity. The database indicated a very high proportion of endemic Chinese plant species in the studied region, accounting for nearly 40% of higher plant species in the region.

**Fig. 1:**
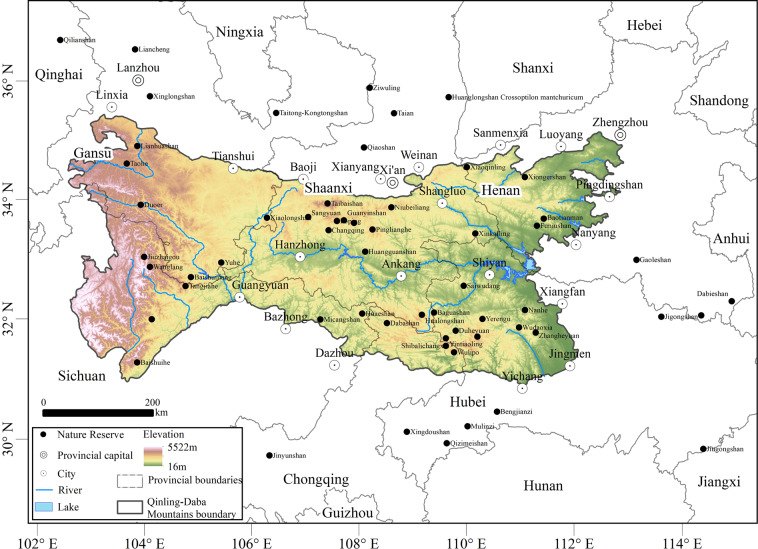
Locations of nature reserves in the Qinling-Daba Mountains.

The database contains information on seed plant species in 58 nature reserves in the Qinling-Daba Mountains and adjacent areas within the range from 102°E–116°E and from 29°N–37°N (Fig. 1), comprising Shaanxi Province, Gansu Province, Henan Province, Sichuan Province, Chongqing Province and Hubei Province and covering an area of approximately 3 × 10^5^ km^2^. Due to the different sizes of nature reserves, we used the central point of each nature reserve to represent its location. All records have geographic coordinates. This dataset is released for noncommercial use only and is licensed under a Creative Commons Attribution 4.0 International License (CC BY 4.0). All publications that use this database should appropriately cite the data and this paper.

The dataset can be used to (i) identify the slope and shape corresponding to the latitudinal gradient pattern of species richness in each order, family and genus and clearly understand the latitudinal gradient pattern of a specific family or genus, which is of great value for targeted afforestation and protection; (ii) evaluate the impact of different life forms and endemism on the latitudinal gradient pattern of overall species diversity using the generalized linear model because the dataset contains attribute information about different life forms (trees, shrubs and herbs) and endemism (whether they are endemic to China or not); and (iii) cluster and comprehensively evaluate plant species in different groups to better understand the survival characteristics and distribution characteristics of Qinling-Daba Mountain forest species, which will be conducive to their further management and protection.

This work aims to integrate our understanding of seed plants in the Qinling-Daba Mountains and adjacent areas, the main body of China’s north–south transition zone. The compilation is provided free of charge to encourage new research on the geographical distribution, ecology and protection of seed plants in China’s north–south transition zone.

## Methods

**Fig. 2:**
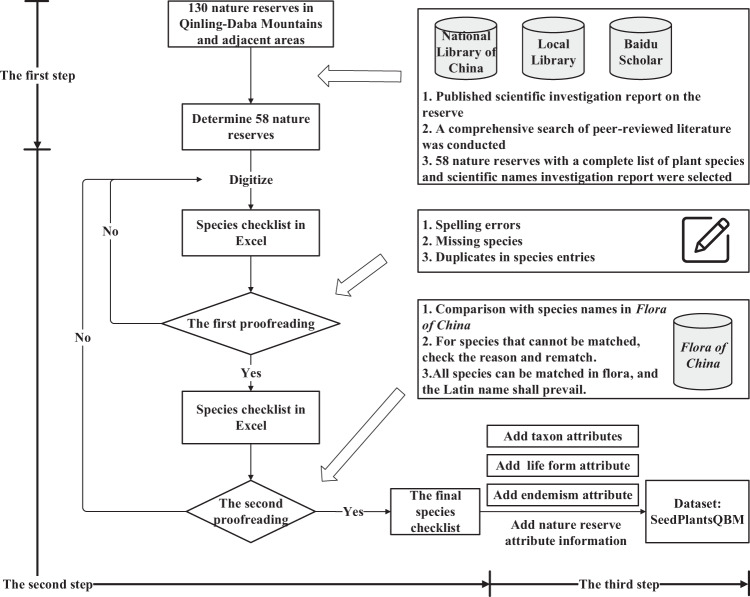
Workflow chart for generating dataset output.

Our data collection and synthesis can be divided into three steps (Fig. 2).

### First – literature search

Through the established reserves published in the “National List of Nature Reserves”^14^, we screened 130 nature reserves in and around the Qinling-Daba Mountains, including at the national, provincial and county levels. Through the National Library of China, the local library at the Institute of Geographical Sciences and Natural Resources Research, Chinese Academy of Sciences and Baidu Scholar, a comprehensive search was conducted for published scientific investigation reports and peer-reviewed documents on the nature reserves. The search results show that the published scientific investigation reports on the nature reserves have the most comprehensive species information. A scientific investigation report is a comprehensive investigation by a scientific investigation team composed of experts from various disciplines in local forestry departments, research institutes and universities to determine the situation of local resources in the nature reserve and further strengthen scientific management. The survey results are comprehensive, reliable and highly authoritative. The species information in the scientific literature is relatively simple and can only be used as a verification aid. According to the species list information in the scientific investigation reports of the nature reserves, we identified 60 nature reserves for which published scientific investigation reports were available, but the species information of two of the nature reserves is incomplete. Therefore, the final checklist of species of the Qinling-Daba Mountains and their surrounding reserves is based on 58 reserves.

### Second – data extraction

We extracted species data and relevant environmental data species by species from the determined scientific investigation report of each reserve, input the data into an Excel spreadsheet, and combined them into a data file. Each data point includes the scientific name of the species and its taxonomic information (order, family, genus, etc.); with very few exceptions, each species can be matched to an accepted name from the *Flora of China* database. The species names were standardized as follows: seed plants mainly refer to the species names and taxonomic trunk in the *Flora of China* database. *Flora of China* is a major database containing the abundant collection of species in China. The anthology includes the scientific names, morphological characteristics, ecological environments, geographical distributions, economic uses and phenological periods of 31142 species of plants belonging to 3408 genera and 301 families in China. The original digitized checklist obtained for seed plant species in the 58 nature reserves of the Qinling-Daba Mountains and adjacent areas has different sources and standards; the taxonomic names were based on those listed in the *Flora of China* database, and any mismatched species names or taxa were manually checked and corrected. The first step in the manual inspection and correction method involved comparing all digitized species with those in the *Flora of China* database and obtaining the name, family, genus and other information of each species with complete consistency. In the second step, in view of the inconsistency of certain words, we manually fuzzy-matched species using the Chinese Field Herbarium (*CFH*) (http://www.cfh.ac.cn) species library to calibrate the digitized original species names (in the case of an incorrect entry or multiple names for a species). In the third step, the corrected species were rematched in the *Flora of China* database, and the species names were obtained. Ultimately, all species could be matched in the flora, and very few were matched using the *CFH species bank*.

The dataset contains collected literature information published from 1994 to 2019, with a total of 96148 records representing 9491 species, 1729 genera, 211 families, and 59 orders. We provide complete taxonomic information for the whole dataset as well as its reference coordinates. The plant species names were obtained from plant checklists characterizing 58 nature reserves in the Qinling-Daba Mountains and its adjacent areas^4,5,8–12,15–66^, and the dataset basically covers all seed plants in the entirety of the Qinling-Daba Mountains and adjacent areas. In addition, we compiled information on the life forms of the relevant and endemic species in China. Plant life forms refer to flora with similar morphological and structural characteristics and represent a convergence phenomenon as well as adaptations to various environments among different taxonomic units^67^. The database includes tree, shrub and herb life forms and includes 1140 tree species, 2046 shrub species and 5742 herb species. Endemic seed plants in China refer to plant species that are naturally distributed only in China^68^. The number of species that are endemic to China in the Qinling-Daba Mountains and its adjacent areas accounts for 38% of the seed plant species in this region.

### Third - database structure

The title of this dataset is “A database of seed plants on taxonomy, geography and ecology in the Qinling-Daba Mountains and adjacent areas”. The database presented here lists 9586 seed plant species and 96148 seed plant records. There are 96148 rows of seed plant records and 23 columns of corresponding attribute information (Table 1) distributed in the Qinling-Daba Mountains and adjacent areas in the database. The 23 columns in the SeedPlantsQBM.csv database and their corresponding unit information are shown in Table 1, with column (A) as code, (B) the name of the nature reserve, (C) the name of the next smallest administrative region than country in which the locality occurs, (D) the name of the country in which the locality occurs, (E) grades of nature reserves, (F) year of the collection, (G) total area of the nature reserve, (H) the geodetic datum in which the coordinates are based, (I) the geographic longitude (in decimal degrees) of the geographic center of the locality, (J) the geographic latitude (in decimal degrees) of the geographic center of the locality, (K) scientific name of the species, (L) scientific name of the genus in which the taxon is classified, (M) scientific name of the family in which the taxon is classified, (N) scientific name of the order in which the taxon is classified, (O) scientific name of the class in which the taxon is classified, (P) scientific name of the phylum in which the taxon is classified, (Q) scientific name of the kingdom in which the taxon is classified, (R) life forms of seed plants, (S) whether the species is endemic to China, (T) whether the nature reserve is located inside or outside the Qinling-Daba Mountains area, (U) references, (V) Scientific name of the species in the International Plant Names Index, (W) Life Sciences Identifier (LSID) of the species in the International Plant Names Index.

**Table 1:** Description of variables contained in the database.

Column	Variable name	Descriptor	Type
A	ID	Code	Numeric
B	NatureReserveName	The name of the nature reserve	Character
C	Province	The name of the next smallest administrative region than country in which the locality occurs	Character
D	Country	The name of the country in which the locality occurs	Character
E	Grade	Grades of nature reserves	Character
F	Year	Year of the collection	Numeric
G	TotalArea/km2	Total area of the nature reserve	Numeric
H	GeodeticDatum	The geodetic datum in which the coordinates are based	Character
I	CentralLongitude	The geographic longitude (in decimal degrees) of the geographic center of the locality	Numeric
J	CentralLatitude	The geographic latitude (in decimal degrees) of the geographic center of the locality	Numeric
K	SpeciesName	Scientific name of the species	Character
L	Genus	Scientific name of the genus in which the taxon is classified	Character
M	Family	Scientific name of the family in which the taxon is classified	Character
N	Order	Scientific name of the order in which the taxon is classified	Character
O	Class	Scientific name of the class in which the taxon is classified	Character
P	Phylum	Scientific name of the phylum in which the taxon is classified	Character
Q	Kingdom	Scientific name of the kingdom in which the taxon is classified	Character
R	Lifeforms	Life forms of seed plants	Character
S	EndemicToChina	Whether the species is endemic to China	Character
T	NatureReserveLocation	Whether the nature reserve is located inside or outside the Qinling-Daba Mountains area	Character
U	References	Scientific investigation reports on species in nature reserves	Character
V	InternationalSpeciesName	Scientific name of the species in the International Plant Names Index	Character
W	LSID	Life Sciences Identifier (LSID) of the species in the International Plant Names Index	Character

## Data Records

All data extracted from the scientific investigation report were input into the above Excel file. The Excel file is named SeedPlantsQBM as a dataset. The dataset contains 96148 records of the distribution of seed plants in the Qinling-Daba Mountains and adjacent areas, including 9491 seed plants, 1729 genera and 211 families, accounting for 39% of the diversity of seed plants in China. The raw data can be found at Figshare^69^. Figshare file 1^69^ summarizes the basic information of each nature reserve and the number of seed plants under specific attributes.

**The terms used in the header of Figshare file 1**^69^ **represent the following meanings:**

**Name:** Name of the nature reserve.

**Province:** Province where the nature reserve is located.

**Grade:** The grade of the nature reserve, including national nature reserves and provincial nature reserves.

**Area:** Total area of the nature reserve in units of km^2^.

**Specific location:** Specific location of the nature reserve.

**Latitude range:** Latitudinal range of the nature reserve.

**Longitude range:** Longitudinal range of the nature reserve.

**Annual mean temperature:** Annual average temperature in the nature reserve in units of °C.

**Coldest month:** Average temperature of the coldest month in the nature reserve, namely, January, in units of °C.

**Hottest month:** Average temperature of the hottest month in the nature reserve, namely, July, in units of °C.

**Annual cumulative precipitation:** Annual cumulative precipitation in the nature reserve, in units of mm.

**Climate type:** Climate types in the nature reserve.

**O:** Order diversity in the nature reserve.

**F:** Family diversity in the nature reserve.

**G:** Genus diversity in the nature reserve.

**SPE:** Species diversity in the nature reserve.

**T:** Tree diversity in the nature reserve.

**S:** Shrub diversity in the nature reserve.

**H:** Herb diversity in the nature reserve.

**E:** Diversity of endemic Chinese species in the nature reserve.

**P:** Proportion of endemic Chinese species in the total number of species in the nature reserve.

## Technical Validation

**Fig. 3:**
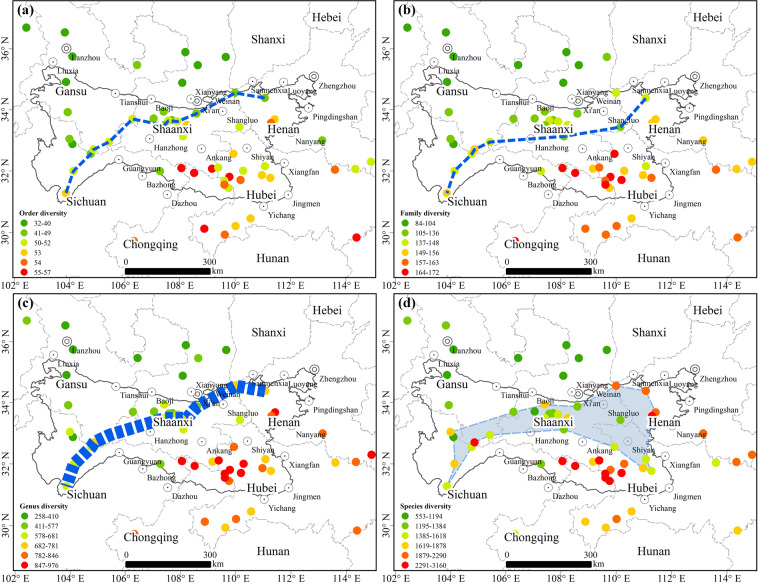
Spatial distribution of diversity of orders, families, genera and species. (**a**) The blue dotted line is basically the dividing line of the order diversity of 50 species. The order diversity to the north of the blue dotted line is lower than 50 species, and the order diversity to the south of the blue dotted line is higher than 50 species. (**b**) The blue dotted line is basically the dividing line of the family diversity of 150 species. The family diversity to the north of the blue dotted line is lower than 150 species, and the family diversity to the south of the blue dotted line is higher than 150 species. (**c**) The thicker blue dotted line is basically the dividing line of genus diversity of 578–681 species. The genus diversity to the north of the blue dotted line is lower than 578 species, and the genus diversity to the south of the blue dotted line is higher than 681 species. (**d**) The blue area is basically the dividing line of species diversity of 1385–1618 species. The species diversity to the north of the blue dotted line is lower than 1385 species, and the species diversity to the south of the blue dotted line is higher than 1618 species.

Each of the 23 key variables can be used for analysis. To validate the dataset, we used five plant-related variables (diversity of order, family, genus, species and species endemic to China) to demonstrate the process of using the dataset for analysis as follows:

(1) For the four variables of plant taxa “order”, “family”, “genus” and “species”, the similarity and difference in spatial distribution pattern of diversity of different taxa in the Qinling-Daba Mountains climate transition zone were analyzed. The spatial distribution pattern of the diversity of the four taxa is shown in Fig. 3, which is increasingly lower from south (low latitude) to north (high latitude). This result is consistent with the classical latitudinal gradient model of plant diversity. The boundary between higher diversity in the south and lower diversity in the north is roughly located in the area of Funiu Mountains in the eastern Qinling-Daba Mountains, Taibai Mountains in the central Qinling-Daba Mountains and Baishui River in the western Qinling-Daba Mountains. However, with the reduction in taxon scale, the spatial distribution pattern of diversity tends to be complex. Orders (Fig. 3a) and families (Fig. 3b) can be divided by lines, while genera (Fig. 3c) need thicker lines, and species (Fig. 3d) can only be divided by polygons. Figure 3 shows that the taxonomic groups of families are more clearly divided, while species can only be divided by staggered bands. Therefore, when dividing the north–south boundary, the family taxon scale is appropriate, whereas the species scale is more appropriate when studying the north–south transition zone.

The dataset can also count the orders, families and genera that appear in 58 nature reserves, indicating that these orders, families and genera are widely distributed in this area, while the orders, families and genera that only appear in a single nature reserve indicate that these taxa are unique to this nature reserve in this area, reflecting their locality and uniqueness, which is helpful to understanding the specific distribution of plants in detail. The relevant statistics are as follows:

**There are 28 orders present in every nature reserve:**

Liliales, Dipsacales, Lamiales, Fabales, Ericales, Poales, Saxifragales, Malpighiales, Malvales, Asterales, Fagales, Gentianales, Geraniales, Ranunculales, Rosales, Solanales, Apiales, Cornales, Brassicales, Caryophyllales, Dioscoreales, Santalales, Myrtales, Asparagales, Celastrales, Sapindales, Alismatales, and Boraginales.

**The order that only appears in one nature reserve is** Petrosaviales, which appears in the Dabashan Nature Reserve in Chongqing.

**There are 51 families present in every nature reserve:**

Liliaceae, Primulaceae, Plantaginaceae, Lamiaceae, Euphorbiaceae, Cannabaceae, Juncaceae, Fabaceae, Poaceae, Elaeagnaceae, Betulaceae, Apocynaceae, Violaceae, Malvaceae, Crassulaceae, Campanulaceae, Asteraceae, Orchidaceae, Polygonaceae, Orobanchaceae, Onagraceae, Gentianaceae, Geraniaceae, Ranunculaceae, Rubiaceae, Rosaceae, Caprifoliaceae, Thymelaeaceae, Apiaceae, Cyperaceae, Cornaceae, Paeoniaceae, Brassicaceae, Amaryllidaceae, Caryophyllaceae, Rhamnaceae, Santalaceae, Asparagaceae, Celastraceae, Sapindaceae, Adoxaceae, Araliaceae, Berberidaceae, Hydrangeaceae, Scrophulariaceae, Convolvulaceae, Urticaceae, Salicaceae, Papaveraceae, Iridaceae, and Boraginaceae.

**There are 15 families that only appear in one nature reserve, as shown in** Table 2.

**Table 2:** Endemic families of the nature reserves in the Qinling-Daba Mountains and surrounding areas.

Nature reserve	Family name
Qilianshan Nature Reserve	Cistaceae, Frankeniaceae
Liancheng Nature Reserve	Ruppiaceae, Cynomoriaceae
Xiaozhaizi Nature Reserve	Dichapetalaceae
Huanglongshan Crossoptilon mantchuricum Nature Reserve	Annonaceae
Jinyunshan Nature Reserve	Aizoaceae, Casuarinaceae, Capparaceae
Saiwudang Nature Reserve	Bromeliaceae, Strelitziaceae, Marantaceae
Micangshan Nature Reserve	Hernandiaceae
Dabashan Nature Reserve	Petrosaviaceae
Mulinzi Nature Reserve	Zamiaceae

**There are 54 genera present in every nature reserve:**

*Patrinia*, *Polygonum*, *Sanicula*, *Plantago*, *Allium*, *Delphinium*, *Euphorbia*, *Juncus*, *Cynanchum*, *Trigonotis*, *Artemisia*, *Sorbus*, *Polygonatum*, *Scutellaria*, *Cirsium*, *Viburnum*, *Ajuga*, *Viola*, *Galium*, *Geranium*, *Salix*, *Epilobium*, *Gentiana*, *Ranunculus*, *Malus*, *Acer*, *Rubia*, *Rosa*, *Torilis*, *Lonicera*, *Adenophora*, *Philadelphus*, *Cornus*, *Paeonia*, *Rhamnus*, *Rumex*, *Carex*, *Thalictrum*, *Asparagus*, *Carpesium*, *Clematis*, *Potentilla*, *Euonymus*, *Eleutherococcus*, *Berberis*, *Spiraea*, *Rubus*, *Populus*, *Vicia*, *Silene*, *Iris*, *Poa*, *Aster*, and *Buddleja*.

There were 225 genera that only appeared in one nature reserve, as shown in Figshare file 2^69^.

**Fig. 4:**
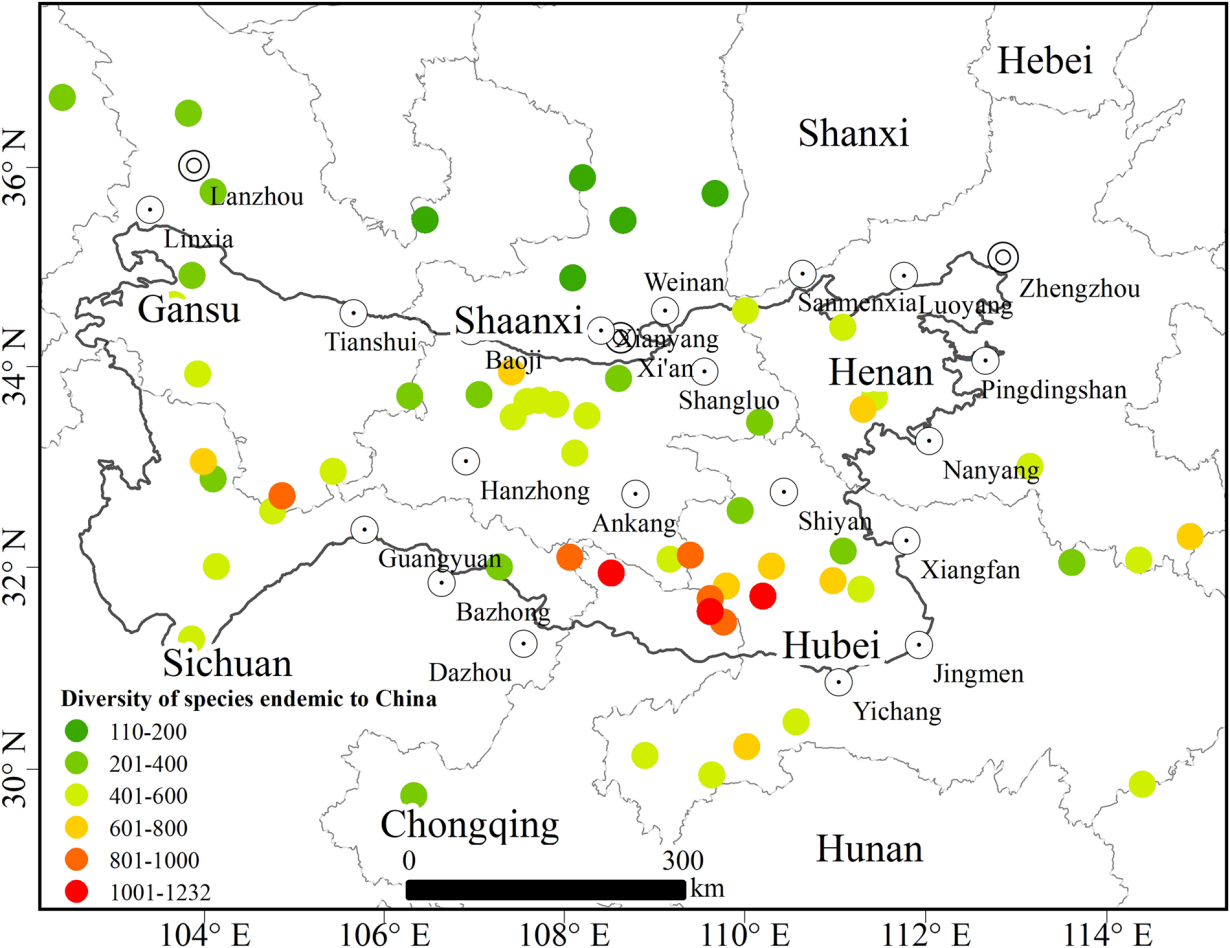
Spatial distribution of diversity of species endemic to China in the Qinling-Daba Mountains and adjacent areas.

**Fig. 5:**
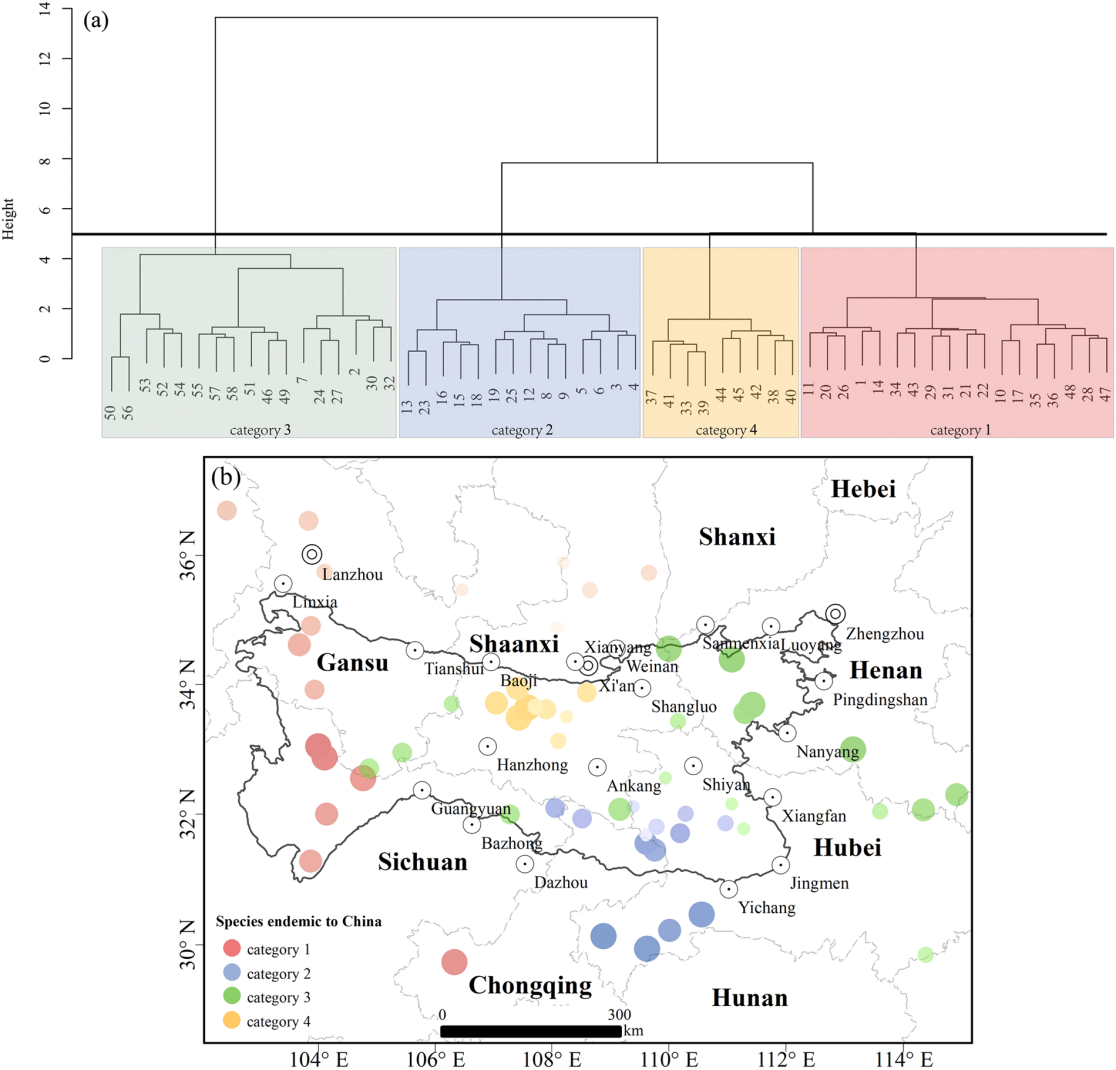
(**a**) Clustering results of Ward’s connection aggregation of species endemic to China in 58 nature reserves. (**b**) Spatial distribution of clustering results of species endemic to China; the larger the dot and the darker the color, the earlier it is merged into this category, and the smaller the dot and the lighter the color, the later it is merged into this category.

(2) For the “species endemic to China” variable of plants, we can see from the diversity distribution pattern of species endemic to China in this region (Fig. 4) that the number of endemic species in the Qinling-Daba Mountains is higher than that of species outside of the region, which reflects the strong transition zone in the Qinling-Daba Mountains. The variables of species endemic to China obtained from the Qinling-Daba Mountains and their surroundings were clustered by the Bray–Curtis dissimilarity measure^70^ and Ward’s minimum variance (the clustering method recommended for plant cluster analysis). The clustering results are shown in Fig. 5a. At the same time, the clustering results are displayed in space. Figure 5b shows that category 3 extends from the east outside the Qinling-Daba Mountains to the Baishuijiang Nature Reserve inside the western Qinling-Daba Mountains, which is consistent with the fact that the Qinling-Daba Mountains are an important ecogeographical “corridor” connecting the east and the west.

## Usage Notes

Our compilation of 58 published field survey data on nature reserves constitutes a meaningful dataset. We suggest that potential dataset users can quantitatively assess the significant impact of forest species diversity on mountain ecosystem characteristics.

In particular, the dataset can be used for the following purposes:

(1) Based on the above datasets, the north–south variation patterns of seed plant order, family, genus and species diversity can be described, and the slopes and shapes corresponding to the latitudinal gradient patterns of species richness in order, family and genus can be counted. The contribution of three higher taxonomic levels (order, family and genus species richness) to the north–south change pattern of species diversity can be calculated. These results can clearly reveal the change pattern of species diversity in each taxon in the northern and southern Qinling-Daba Mountains, which is of great value for targeted afforestation and protection. For example, the taxa Rosales, Lamiales, Lamiaceae, Rosaceae, *Rubus*, *Viburnum* and *Ilex* are more diverse in the southern and less diverse in the northern Qinling-Daba Mountains, indicating that the southern environment is more suitable for the survival of these taxa. In artificial afforestation and other projects, more plants in this subgroup could be planted in the southern Qinling-Daba Mountains to increase the survival rate of plants; Ephedrales, Podocarpales, Ephedraceae, Podocarpaceae, *Potentilla* and *Astragalus* are less diverse in the southern and more diverse in the northern Qinling-Daba Mountains, indicating that the northern environment is more suitable for the survival of these taxa. Thus, more plants in these taxa can be planted in the northern Qinling-Daba Mountains. Therefore, understanding the specific development and distribution patterns of species in different plant taxa can provide detailed guidance for sustainable forest management. The use of these specific plant development and survival distribution characteristics is conducive to afforestation and species protection. A better understanding of the survival characteristics and distribution characteristics of forest species in the Qinling-Daba Mountains will be conducive to their further management and protection.

(2) The change patterns of different life forms (trees, shrubs and herbs) along latitude gradients can be analyzed. The community structure of different life forms not only improves the ability of the community to use environmental resources such as sunlight but also creates a variety of habitat and food conditions for animals. Therefore, understanding the latitudinal gradient change patterns of trees, shrubs and grasses can improve the efficiency and accuracy of efforts to manage forest species diversity in the Qinling-Daba Mountains. For example, tall trees are suitable for Crested Ibis nesting, dense bamboo forests in mountains provide food for giant pandas, and dense forests in mountains provide safe habitats for golden monkeys.

(3) At the same time, the spatial distribution pattern of plant endemism (species endemic and nonendemic to China) can be clarified. Whether a plant is endemic to a given area is an important consideration in the setting of nature reserves. Understanding the distribution of species endemic to the Qinling-Daba Mountains in China provides important information for determining the priority of protected areas of these organisms, which is conducive to the implementation of protection actions and promotes the effective distribution of these efforts.

The above study and other related studies using the dataset will provide a basis for forestland management, the establishment of nature reserves and species protection. Especially in the Qinling-Daba Mountains (a complex climate transition zone), combined with the change patterns of the plant latitude gradient in different groups, biodiversity protection measures can be more efficient and targeted, which is helpful for understanding the impact of the ecological environment on forest ecosystems.

These distribution patterns and latitude gradient patterns are only used to validate the dataset and explain a few ways in which potential users could use the dataset.

## Data Availability

No specific codes were used to produce the data presented.
